# Complete study demonstrating the absence of rhabdovirus in a distinct Sf9 cell line

**DOI:** 10.1371/journal.pone.0175633

**Published:** 2017-04-19

**Authors:** Yoshifumi Hashimoto, Daniel Macri, Indresh Srivastava, Clifton McPherson, Rachael Felberbaum, Penny Post, Manon Cox

**Affiliations:** Protein Sciences Corporation, Meriden, Connecticut, Unites States of America; Ecole des Mines d'Ales, FRANCE

## Abstract

A putative novel rhabdovirus (SfRV) was previously identified in a *Spodoptera frugiperda* cell line (Sf9 cells [ATCC CRL-1711 lot 58078522]) by next generation sequencing and extensive bioinformatic analysis. We performed an extensive analysis of our Sf9 cell bank (ATCC CRL-1711 lot 5814 [Sf9^L5814^]) to determine whether this virus was already present in cells obtained from ATCC in 1987. Inverse PCR of DNA isolated from Sf9 ^L5814^ cellular DNA revealed integration of SfRV sequences in the cellular genome. RT-PCR of total RNA showed a deletion of 320 nucleotides in the SfRV RNA that includes the transcriptional motifs for genes X and L. Concentrated cell culture supernatant was analyzed by sucrose density gradient centrifugation and revealed a single band at a density of 1.14 g/ml. This fraction was further analysed by electron microscopy and showed amorphous and particulate debris that did not resemble a rhabdovirus in morphology or size. SDS-PAGE analysis confirmed that the protein composition did not contain the typical five rhabdovirus structural proteins and LC-MS/MS analysis revealed primarily of exosomal marker proteins, the SfRV N protein, and truncated forms of SfRV N, P, and G proteins. The SfRV L gene fragment RNA sequence was recovered from the supernatant after ultracentrifugation of the 1.14 g/ml fraction treated with diethyl ether suggesting that the SfRV L gene fragment sequence is not associated with a diethyl ether resistant nucleocapsid. Interestingly, the 1.14 g/ml fraction was able to transfer baculovirus DNA into Sf9^L5814^ cells, consistent with the presence of functional exosomes. Our results demonstrate the absence of viral particles in ATCC CRL-1711 lot 5814 Sf9 cells in contrast to a previous study that suggested the presence of infectious rhabdoviral particles in Sf9 cells from a different lot. This study highlights how cell lines with different lineages may present different virosomes and therefore no general conclusions can be drawn across Sf9 cells from different laboratories.

## Introduction

*Spodoptera frugiperda* (Sf) cell lines, such as Sf21, Sf9, and *expres*SF+^®^ (SF+), BTI-TN5B1 (High Five) cells from the *Trichoplusia ni* cabbage looper, and BmN cells from the *Bombyx mori* silkworm are widely being used in baculovirus research and for recombinant protein production using the baculovirus expression vector system (BEVS) [1][2][3][4][5][6]. The use of these cell lines has led to the discovery of persistent infections of RNA viruses [7] [8] [9]. For example, a nodavirus was found in High Five cells [7] that were infected with a recombinant baculovirus containing a hepatitis E capsid gene and enhanced nodavirus proliferation was observed in infected cells. with the recombinant baculovirus. The nodavirus forms 35 nm virus particles containing two RNA segments, similar to those of the flock house virus [7].

Recent advances in next generation sequencing (NGS) technology have enabled the discovery of covert and previously un-described insect RNA viruses [10][11][12] and metagenomic analysis has been used to identify new viruses and endogenous viral elements (EVEs) associated with economically important insects [13][14]. Although the assembly of RNA virus genomes from NGS data resulted in a more complete sequence for 5’-untranslated regions than the use of 5’-RACE [15][16][17]., Liu et al. [10] have pointed out that analysis of NGS data often results in identification of contigs with sequence similarity to viral sequences through BLAST, but the results may actually come from EVEs. Unfortunately, there is no reliable way to distinguish between real viral sequences and EVEs from NGS data analysis without extensive experimentation.

Recently, using NGS, Ma et al. [1] reported the identification of a novel rhabdovirus sequence in Sf9 cells (SfRV, Sf9 cells from ATCC [CRL-1711, lot 58078522]). This SfRV RNA is comprised of five genes, N-P-M-G-L, flanked by transcription motifs, such as polyA signals and transcription start sites within intergenic regions, which are typical for the majority of rhabdoviruses. However, SfRV also possessed a number of unique characteristics including one small gene, X, located between genes G and L that encodes a 109 amino acid peptide. Similar genome organization, N-P-M-G-U1-L, and conserved transcription motifs have recently been reported in two mosquito rhabdoviruses, Arboretum virus (ABTV) and Puerto Almendras virus (PTAMV)[18]. A limited region of the SfRV L protein showed similarity to domain III of rhabdovirus RNA-dependent RNA-polymerase and corresponding region in the Taastrup virus, a plant rhabdovirus transmitted by a leafhopper[19]. Also, a search for SfRV RNA sequences in the public sequence database indicated a high similarity of the SfRV M gene to a mRNA clone derived from BmN cells (AK377209.1). However, leader and trailer sequences including ABTV and PTAMV [18], that are believed to function in transcription and replication of rhabdoviruses [20][21] were not found in SfRV. Moreover, the morphology of purified SfRV particles was inconsistent in shape and size compared to the typical bullet and bacilli-like morphology of rhabdovirus particles[22][23].

In this report, we performed an extensive analysis of an earlier Sf9 cell lot (ATCC, CRL-1711, lot 5814 [Sf9^L5814^]) using virological methods and public sequence databases to determine the presence of an SfRV entity.

## Materials and methods

### Cell line

Sf9 cell line CRL-1711 lot 5814, Sf9^L5814^, was obtained from ATCC (Manassas, VA) in 1987 [24][25]. Sf9^L5814^ cells were maintained in shake flasks with complete TNM-FH insect medium (Sigma-Aldrich, St. Louis, MO) containing 0.1% Pluronic F68 (Thermo Fisher Scientific, Waltham, MA).

### SfRV sequence search in public databases

Public sequence databases used in this study are a draft Sf whole genome shotgun sequence (GenBank, JQCY00000000.2), Bm strain p50T (= Dazao) whole genome shotgun sequence (GenBank, BABH00000000.1), and EST database (as of July 2015) in GenBank and SPODOBASE (http://bioweb.ensam.inra.fr/spodobase/). SfRV RNA sequence was retrieved from GenBank KF947078.1.

### RT-PCR of Sf9^L5814^ total cellular RNA

Total cellular RNA was extracted from Sf9^L5814^ cells (5 x 10^6^ cells) in a late growth phase culture (2.0–3.0 x 10^6^ cells/ml, 98% cell viability) by using the miRNeasy mini kit (Qiagen, Valencia, CA). One-step RT-PCR was performed on total cellular RNA using SuperScript One-Step RT-PCR with Platinum^®^ Taq (Thermo Fisher Scientific, Waltham, MA). Fourteen primer sets covering the SfRV RNA sequence between positions 41 and 13,449, were used (S1 Table).

### Inverse PCR

Genomic DNA was isolated from 5 x 10^6^ Sf9^L5814^ cells using the DNeasy Blood & Tissue Kit (Qiagen, Valencia, CA). Ten μg of genomic DNA was digested with *Bgl*II, *Eco*RI, or *Nco*I (New England Biolabs [NEB], Ipswich, MA) at 37°C overnight. Digested DNA was extracted with 25:24:1 phenol-chloroform-isoamyl alcohol, precipitated in 70% ethanol, collected by centrifugation, and suspended in TE buffer. One μg of digested DNA was subjected to self-ligation in a 500 μl reaction containing T4 DNA ligase (NEB, Ipswich, MA) at 16°C overnight. The ligation mixture was used for in an inverse PCR followed by nested PCR. The ligation mixture was extracted with 25:24:1 phenol-chloroform-isoamyl alcohol, precipitated in 70% ethanol, and suspended in 10 μl TE buffer, then inverse PCR was performed. The inverse PCR product was diluted 10^3^-fold with TE buffer and subjected to nested PCR. Inverse PCR and nested PCR primers were designed to detect 17 loci on SfRV RNA (S2 Table). The dominant nested PCR product was purified, cloned using the Zero Blunt TOPO PCR cloning kit (Thermo Scientific, Waltham, MA) and sequenced using SfRV specific primers. The resulting sequence was subjected to a BLAST search against the draft Sf whole genome shotgun sequencing project (Genbank, JQCY00000000.2).

### Sucrose density gradient purification

Culture supernatant in a late growth phase was centrifuged at 300 x g for 10 min, filtered using 0.45 μm filter discs, and the filtrate was concentrated by centrifugation (Beckmann 70 Ti rotor at 41,300 rpm for 2 hr). The pellet was suspended in phosphate buffered saline (PBS), pH 7.4 (Thermo Scientific, Waltham, MA), loaded on a 10 to 60% (w/v) sucrose gradient in PBS, and centrifuged (Beckmann SW 41 Ti rotor at 28,000 rpm overnight). One ml fractions were collected, diluted in PBS, and centrifuged (Beckmann SW 41 Ti rotor at 28,000 rpm for 1.5 hr). The pellets were suspended in 200 μl of nuclease free water, and RNA was extracted by miRNeasy Mini kit (Qiagen, Valencia, CA). RNA was used in qRT-PCR for the detection of SfRV RNA. A single white band was observed at a density of 1.14g/mL (1.14 fraction).

### qRT-PCR

ABI 7500 fast Real Time PCR system (Applied Biosystems, Foster City, CA) was used for qRT-PCR assays to detect baculovirus p10 and SfRV L gene transcripts using the Power SYBR^®^ Green RNA-to-CT™ 1-Step Kit. The primers are listed in S3 Table.

### Electron microscopy

Negative staining of the 1.14 fraction was carried out by Paragon Bioservices (Baltimore, MD). In brief, 100 μl suspension of the 1.14 fraction was diluted 1:10 or 1:30 with distilled water and 5 μl of sample was loaded on a grid, which was either incubated for 1 min or processed immediately. The grid was washed twice with distilled water and the sample was stained with 1% uranium acetate.

### SDS-PAGE and LC-MS/MS

The 1.14 fraction was analyzed by SDS-PAGE using a Novex 4–12% Tris-Glycine gel (Thermo Scientific, Waltham, MA) according to the manufacturer’s instructions. The gel was stained with Coomassie brilliant blue, and gel pieces containing major protein components of the 1.14 fraction were excised and submitted to The Yale Keck MS & Proteomics Resource to perform LC-MS/MS for protein identification. The major protein component from each gel piece was identified by an NCBI nr database search, which were searched in an exosomal marker protein database, ExoCarta (http://exocarta.org/query.html).

### Transfection of Sf9^L5814^ cells with baculovirus DNA using the 1.14 fraction as transfection reagent

Sf9^L5814^ cells were transfected with baculovirus DNA using the 1.14 fraction as a biological carrier. Dimethyldidecylammonium bromide and dioleoylphosphatidyl ethanolamine were used as control transfection reagents [26]. In brief, 2 μg of baculovirus DNA was mixed with 10 μl of 1.14 fraction or control reagents, and the mixture was incubated at room temperature for 30 min. The mixture was combined with 1 ml of TNM-FH medium, added to a 6 cm diameter culture dish containing 0.5 x 10^6^ cells and incubated at room temperature for 4 hr with intermittent rocking. Cells were then rinsed and incubated using TNM-FH medium at 27°C for 5 days. The supernatant from the transfected cell culture was used for RNA extraction (Qiagen miRNA mini kit [Qiagen, Valencia, CA]). Total RNA was treated with DNase I and analyzed by qRT-PCR using p10 gene specific primers (S3 Table). Baculovirus infection was confirmed by the presence of polyhedra.

### Diethyl ether treatment of the 1.14 fraction of Sf9^L5814^ culture supernatant

The 1.14 fraction from Sf9^L5814^ culture supernatant was treated with an equal volume of diethyl ether, followed by centrifugation (Beckmann SW 41 Ti rotor at 28,000 rpm for 1.5 hr) [27][28]. RNA was extracted from the supernatant and pellet and subjected to qRT-PCR as described above. A wild-type extracellular baculovirus was used as a control enveloped virus.

## Results

### SfRV and SfRV-like sequences are integrated in the Spodoptera frugiperda genome

We performed a BLAST search for SfRV sequences in the draft whole genome shotgun sequence of the Sf21 *Spodoptera frugiperda* cell line ([29]; GenBank, JQCY00000000.2). Three Sf21 genome sequence scaffolds showed similarity to a portion of the SfRV P, G, and L genes with identities of 75, 74, and 71% in nucleotide sequence and 85, 82, and 82% in protein sequence, respectively (Table 1). These Sf21 genome sequences will be referred to as SfRV-like sequences in this study. The SfRV P gene-like sequence encodes a C-terminal region of the SfRV P protein with a size of 269 amino acids. The SfRV G gene-like sequence encodes an N-terminal region of the SfRV G protein with a size of 378 amino acids. The SfRV L gene-like sequence encodes the middle region of the SfRV L protein with a size of 443 amino acids, which corresponds to a conserved domain of *Mononegavirales* (pfam00946, E value = 6.04e-58) and Wuhan Ant virus (GenBank KM817645, E value = 2e-107) RNA dependent RNA polymerase (RdRp).

**Table 1:** BLAST analysis of SfRV RNA (GenBank, KF947078) against Sf21 cell genome shotgun sequence (GenBank, JQCY00000000.2).

QUERY: Start..end in SfRV RNA (gene, position in SfRV RNA)	SUBJECT: Sf21 shotgun sequence		Identity, %	
	Hit: Start..end	Scaffold ID	Nucleotide	Amino acid
2031..2867 (P, 1726..2781)	10455..9619	9702	71	85
4110..5224 (G, 4140..5972)	70..1184	5970	74	82
8543..9801 (L, 6833..13255)	13664..12406	7162	75	82

Inverse PCR followed by nested PCR was used to evaluate whether SfRV sequences were also integrated in the genome of our Sf9^L5814^ cells, a sub-cell line of Sf21. Four clones with following structure were identified: nested PCR primer-SfRV RNA-Sf9 genome-SfRV RNA-nested PCR primer confirming integration of the SfRV into the Sf9 genome (Table 2). Two clones contained sequences corresponding to the N-terminal region of the SfRV N protein, one clone contained a sequence corresponding to the C-terminal region of the SfRV P protein, and one clone contained a sequence corresponding to a middle region of the SfRV L protein. Unlike the SfRV-like sequences in the Sf21 genome (Table 1), no SfRV G-like sequences were obtained by inverse/nested PCR of Sf9 ^L5814^ genomic DNA; instead, two SfRV N-like sequence clones were obtained. The sequences of the four inverse PCR clones showed high similarity to the SfRV sequence (amino acid and nucleotide sequence identities, >99%). Similar to the SfRV L-like sequence in the Sf21 genome, the inverse PCR clone sequence from the Sf9 ^L5814^ genome contained a large portion of the *Mononegavirales* RdRp (pfam00946, E value = 3.98e-89) and the *Mononegavirales* mRNA-capping region V (pfam 14318, E value = 4.44e-18) domains. The four inverse PCR clones represent 29.5% of the length of SfRV RNA.

**Table 2:** Chimeric sequence of SfRV and Sf genome in four inverse PCR clones obtained from Sf9 DNA.

Inverse PCR clone, size (kb)	SfRV sequence found (start..end of SfRV genome)	Identity, %		Sf21 shotgun sequence	
		Nucleotide	Amino acid	Scaffold ID	SfRV sequence insertion position in scaffold
Bg.8 (4.0)	3..293	99	99	277	53272/53270
Nco.2.1 (5.0)	17..296	99	99	34	33223/33222
Eco.6 (1.7)	2074..2971	99	99	4119	9999/11231
Nco.7.7 (4.3)	7839..10539	99	99	900	163834/163835

### EST clones carrying SfRV-like and SfRV sequences are found in Spodoptera frugiperda and other insect species

**Fig 1:**
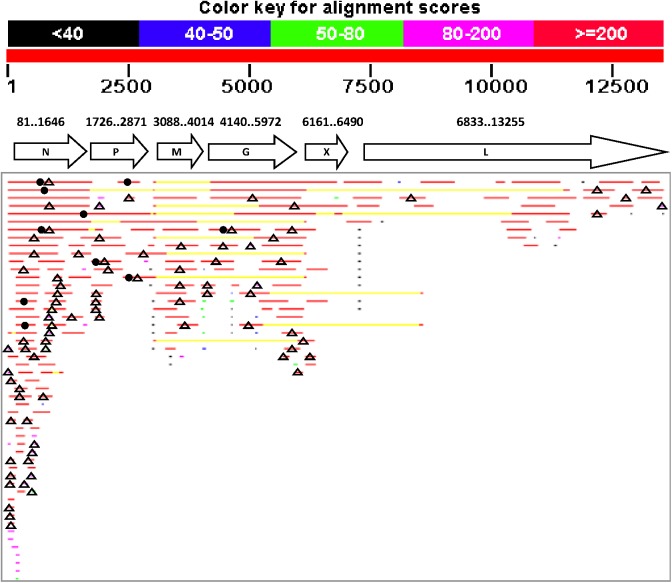
BLAST search of SfRV RNA sequence against the Sf21 draft whole genome shotgun sequence (GenBank, JQCY00000000.2). SfRV genes and their position on the genome are presented under the scale bar. Sf21 genome DNA sequences showing homology to SfRV RNA are aligned on the map; open triangle, anti-sense (SfRV genome RNA strand); closed circle, chimera with Sf21 DNA sequence.

A second BLAST search for SfRV sequences was performed against the Sf EST database in SPODOBASE to assess the use of canonical transcription motifs and to map EST clones onto the SfRV genome. Results showed 228 EST clone hits with E values ≤ 4 x 10^−4^, including two hits from the Sf9L EST library (Sf9 cell line sequence) and 226 hits from the Sf9LR EST library (Sf9 cell line sequences from G. Rohrmann) (Fig 1, S4 Table). 110 EST clone sequences showed a very high similarity (E value = 0) to SfRV RNA and 118 showed an E-value ≤4 x 10^−4^. The 228 positive EST clones represented 0.2% of the cDNA reads in Sf9L+Sf9LR EST libraries. EST clones aligned onto the SfRV genome map showed that the number of clones decreased proportionally from 3’ to 5’ (Fig 1). Approximately half of these EST hits contained a nucleotide gap(s) in the sequence when aligned to the SfRV genome sequence, which revealed that these EST clones contain sequence that is out of frame. Approximately one third of the EST clones contained the negative sense SfRV sequence (SfRV genome sense), confirming incorporation of the SfRV genome sequence into the insect genome. Eight EST clones contained chimeric SfRV sequences, which spanned the SfRV genome map. Ten EST clones contained chimeric SfRV like-Sf genome sequences. The 3’ ends of most of the EST clone sequences did not align to putative polyA signal sequences of SfRV genes.

**Fig 2:**
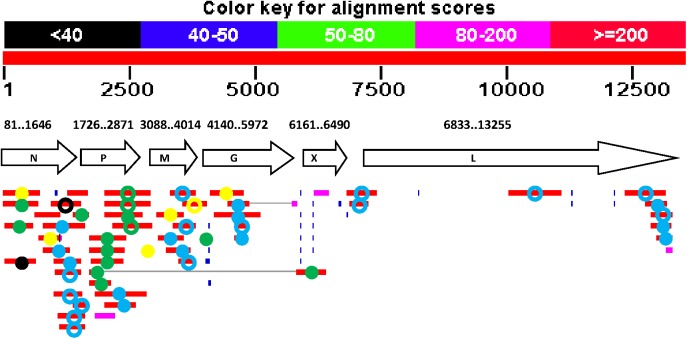
BLAST search of SfRV RNA sequence against GenBank EST database. EST clone sequences showing homology to SfRV RNA are aligned on the map and represented by colored circles; yellow circle, *Bombyx mori* (Bm5 or BmN cells, or Nnor); green circle, BmNPV infected BmN cells; blue circle, *Heliothis virescence*; black circle, *Aphid gossippi*; closed circle, sense strand sequence; open circle, anti-sense strand sequence.

A third BLAST search using the SfRV sequence against the GenBank EST libraries showed hits for 51 EST clones, including 15 from an EST library of BmN cells infected with BmNPV, 5 from an EST library of BmN and Bm5 cells, 29 from an EST library of *Heliothis virescence* (Hv) larva, and 2 from an EST library of *Aphis gossyppi* (Ag) (Fig 2, S5 Table). Among the 51 EST clones, 19 contained negative sense SfRV sequences. All EST clones from the Bm cell lines, with and without BmNPV infection, showed high similarity to SfRV RNA (DNA sequence identity = 93–100%); however, EST clone sequences from libraries of Hv and Ag showed DNA identities between 69 and 81%. Similar to the BLAST search results of the SfRV sequence against the SPODOBASE EST libraries, the 3’ ends of the EST clones did not align to putative polyA signal sequences of SfRV genes. One EST clone from BmNPV infected BmN cells contained a chimeric SfRV sequence, and the number of EST clones again decreased proportionally from 3’ to 5’ on the SfRV map (Fig 2).

### 320 nt region is missing in SfRV RNA from Sf9^L5814^ cells

**Fig 3:**
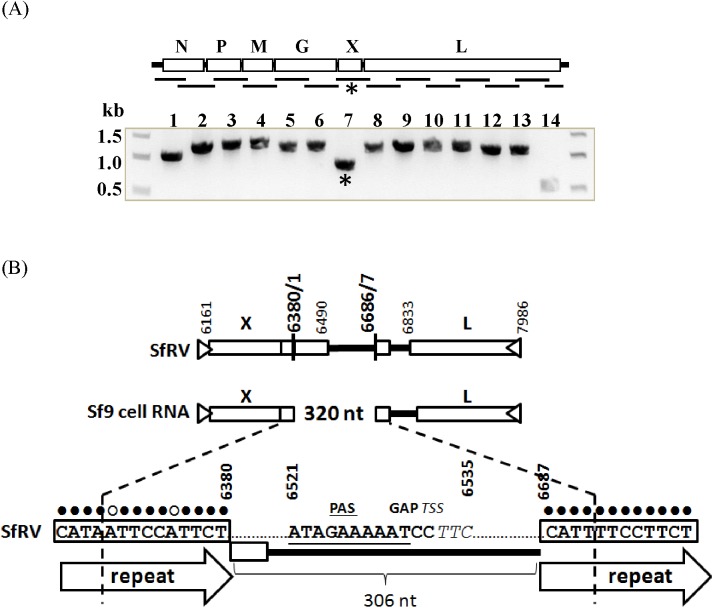
(A). RT-PCR of total RNA from Sf9 cells. Primers were designed to amplify 14 target sequences that cover 99.1% of the reported Sf-rhabdovirus RNA in an overlapping manner as indicated by the gene schematic. Sizes of 13 target sequences ranged from ~1.0 to 1.3 kb (lanes 1–13) and one target sequence was ~0.5 kb (lane 14). The sizes of amplicons matched the expected sizes of target sequences except for one amplicon (*, lane 7), which was amplified by primers targeting bp 6965–7134. (B). Sequencing of the amplicon revealed a deletion of 320 bp at position 6371–6690 in Sf-rhabdovirus RNA. The 320 bp deletion contains the 3’ region of ORF-X and a part of the intergenic region between ORF-X and ORF-L that includes rhabdovirus conserved transcription motifs for the X and L genes.

Total RNA from Sf9^L5814^ cells was analyzed by RT-PCR to evaluate whether a complete SfRV RNA genome sequence was present. Fourteen overlapping primer sets covering 98.6% of the SfRV RNA sequence were used. Forward and reverse primers were designed to cover 1.0 to 1.3 kb regions of SfRV RNA, except for the primer set to detect the 5’ end region of SfRV RNA that would amplify only 558 bp (primers 12891F and 13449R) (S1 Table). All primer sets yielded PCR products of the expected size, except the primer set covering the region between SfRV RNA positions 5965 and 7134 (primers 5965F and 7134R) (Fig 3A), which was smaller than expected. Sequencing of this PCR product revealed a 320 nt deletion that encompassed the C-terminal region of the SfRV X gene and the polyA-gap-transcription start site, which is a conserved rhabdovirus transcriptional motif present in the intergenic region between genes X and L (Fig 3B). Also, fourteen nucleotide imperfect repeats (CATAA[T]TTCCA[C]TTCT) containing the junctions of the 320 nt deletion and flanking sequences were identified (Fig 3B). To ensure that this 320 nt deletion was not an artifact produced during cDNA synthesis (e.g., due to RNA template secondary structure), RT-PCR was performed using primers specific for the deleted region in combination with flanking primers. cDNA synthesis at an increased temperature (60°C) was also tested. Both methods confirmed the absence of the 320 nt sequence in total RNA from Sf9^L5814^ cells (data not shown). Our sequence results differ somewhat from the published SfRV sequence and show three mutations in the N protein gene, two of which were silent; one silent mutation in the M protein gene; nine mutations in the L protein gene, four of which were silent; one mutation in the intergenic region between the M and P genes; and one mutation in the 5’ un-translated region (S6 Table).

### Analysis of the 1.14 sucrose gradient fraction

#### Structural analysis by electron microscopy

**Fig 4:**
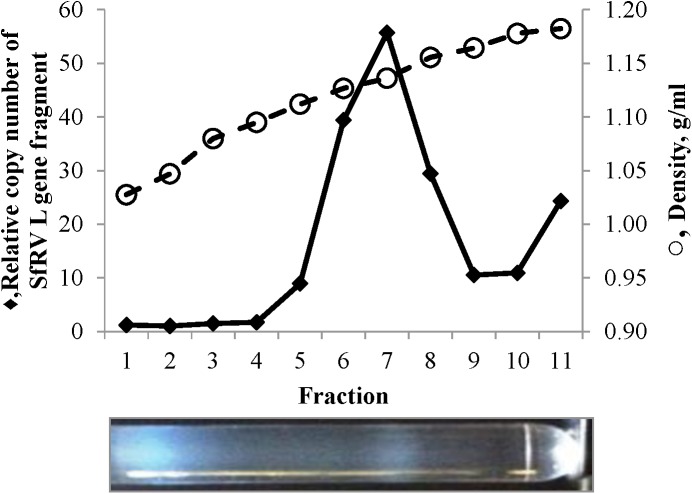
qRT-PCR of sucrose density gradient fractions using SfRV L gene primers. Ct values were normalized to that of fraction 2 and are represented as a relative copy number of target SfRV L gene sequence. The photo shows the sucrose density gradient prior to fractionation. Left to right: top to bottom of the gradient.

**Fig 5:**
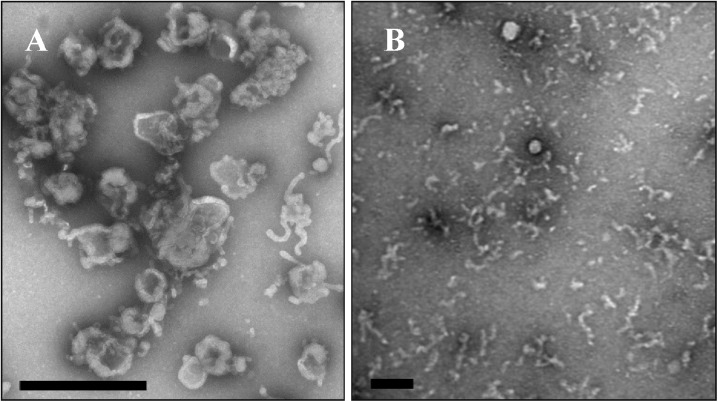
Electron microscopy of the 1.14 g/ml fraction. Bar indicates 100 μm in length. A, no incubation after loading the 1.14 fraction (diluted with distilled water) onto a grid. B, 1 min incubation after loading. Both grids were washed twice with distilled water and sample was stained with 1% uranyl acetate.

A sucrose density gradient of filtered and concentrated Sf9^L5814^ culture supernatant showed a single white band at a density of 1.14 g/ml (1.14 fraction) (Fig 4). qRT-PCR analysis on RNA isolated showed that the L gene sequence was concentrated in the 1.14 fraction. This fraction was further analysed by electron microscopy. Negative staining of an ultracentrifuge pellet of this fraction revealed the absence of typical rhabdovirus structures, such as bullets or bacilliform particles greater than 100 nm in length. Rather, the fraction contained particulate and amorphous debris consisting of membranous structures (Fig 5A). A large portion of the particulate objects disappeared and amorphous debris increased when the 1.14 fraction was incubated on the grid for 1 min and then processed for washing and staining (Fig 5B). These structures were absent in complete TNM-FH media (data not shown).

### The 1.14 fraction contains an incomplete set of SfRV proteins and a collection of exosome marker protein homologs

#### Protein analysis

**Fig 6:**
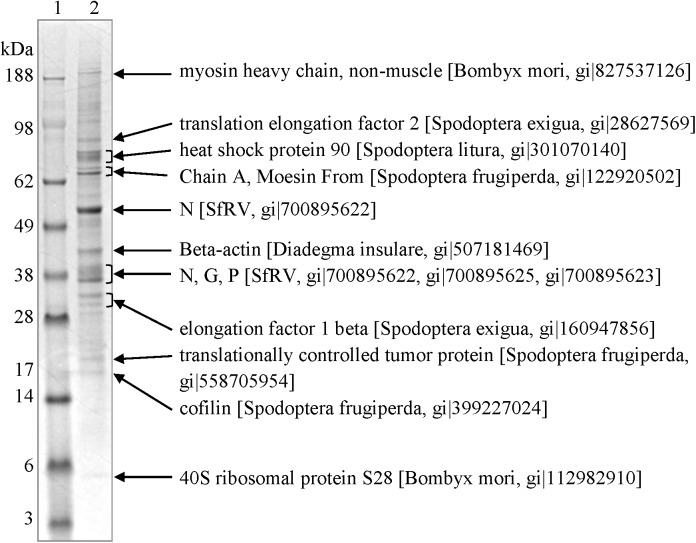
Identification of major protein components in the 1.14 g/ml sucrose density gradient fraction of Sf9 culture supernatant by LC MS/MS analysis. Eleven groups containing up to 3 stained protein bands were analyzed. The major protein identified per band is listed on the right.

The 1.14 fraction was analyzed by SDS-PAGE (Fig 6). The profile in the stained gel showed diverse proteins with molecular masses ranging from 5 to 200 kDa. The two major protein components were 38 kDa and 98 kDa in size. Proteins similar in mass to putative SfRV proteins (N, 58.4 kDa; P, 42.0 kDa; M, 35.0 kDa; G, 69.9 kDa; X, 12.6 kDa; L, 244.3 kDa) were not evident. Eleven SDS-PAGE gel slices containing 17 major protein bands in the 1.14 fraction were analyzed by LC MS-MS to identify the proteins. Fig 6 lists the major proteins identified in each group based on their molecular mass, percent coverage by BLAST search, and emPAI value by MS analysis. These and other proteins identified in the gel pieces are summarized in S7 Table. SfRV N protein was identified as a major component of the 1.14 fraction with a high emPAI value (27.05). SfRV P and partial N and G proteins were identified in the gel piece near the 38 kDa marker. No SfRV proteins were identified in the remaining nine gel pieces. The non-SfRV proteins were identified as homologues of the exosome proteins from *Drosophila* and other organisms by searching against the ExoCarta database.

### Diethyl ether treatment of the 1.14 fraction indicated absence of enveloped virus

The 1.14 fraction was treated with diethyl ether to evaluate whether the SfRV proteins identified by LC MS-MS and the L-gene sequence were derived from an enveloped virus particle. Diethyl ether will solubilize any potential viral envelopes and membranous structures, but the nucleocapsid of an enveloped virus particle will remain intact. After treatment, the fraction was ultra-centrifuged to pellet any potential nucleocapsids, and RNA extracted from both pellet and supernatant was analyzed by qRT-PCR using SfRV L gene specific primers. The distribution ratio of SfRV L gene sequence copy number between supernatant and pellet was 1 to 0.06. Thus the majority of the SfRV L sequence identified in the 1.14 fraction is not found in the ultracentrifuged pellet after diethyl ether treatment, whereas in contrast, a positive control containing enveloped baculovirus gave a distribution ratio of 1 to 82 (supernatant to pellet) for the baculovirus p10 gene sequence. These data indicate that the SfRV N, P, and partial N and G proteins identified by LC-MS/MS do not form an enveloped virus particle containing an SfRV L sequence.

### The 1.14 fraction is competent as a transfection reagent, suggesting the presence of functional exosomes

LC-MS/MS analysis revealed that major proteins present in the 1.14 fraction are homologs of exosome marker proteins. Exosomes have been shown to function not only as vehicles for cellular components in culture, but also as carriers of foreign nucleic acids in vertebrate cell transfection. We tested whether the1.14 fraction could transfer a baculovirus DNA into Sf9^L5814^ cells. qRT-PCR was performed to confirm expression of the baculovirus p10 gene in the transfected cells. The results showed that the relative copy numbers of p10 transcript were 3.65 ± 0.05 forPBS, 1.50 ± 0.08 for the 1.14 fraction alone, 0 for baculovirus DNA alone, 1.38 x 10^5^ ± 4.33 x 10^3^ for baculovirus DNA plus the 1.14 fraction, and 1.21 x 10^6^ ± 3.20 x 10^5^ for baculovirus DNA plus lipid reagents (positive transfection reagent control). Production of wild-type baculovirus occlusion bodies was confirmed in the cultures incubated with transfection supernatant from baculovirus DNA plus the 1.14 fraction and in baculovirus DNA plus lipid transfection reagents, but not with baculovirus DNA, 1.14 fraction, or lipid reagents alone (not shown). These results suggest that the 1.14 fraction contains functional exosomes, and that they can deliver baculovirus DNA into Sf9^L5814^ cells.

## Discussion

A BLAST search for SfRV sequences against multiple public sequence databases and inverse PCR of Sf9^L5814^ DNA demonstrated the presence of SfRV-like sequences (Table 1) and SfRV sequences (Table 2) integrated in the Sf9^L5814^ genome. Maghodia et al. [30] independently searched an Sf genome database and identified SfRV-like sequences by bioinformatic analysis, as well as experimentally confirmed the presence of SfRV-like sequences and transcripts in Sf9 cells. Our SfRV sequence search against the EST database also identified SfRV-like and SfRV sequences (S4 and S5 Tables). Additionally, our inverse/nested PCR analysis of Sf9^L5814^ DNA using SfRV specific primers showed three sequences that are nearly identical to the SfRV sequence in the database (>99% for nucleotide and protein sequence, Table 2).

Bioinformatic analysis and alignment of the EST clone sequences with the SfRV genome map indicated a complex feature of transcription of the SfRV sequence (Figs 1 and 2). Unexpectedly, the EST clone sequences from Sf and Bm EST clones are nearly identical to the SfRV sequence, despite originating from different species. Sf EST clone sequences encode both SfRV genome (-) and (+) sense sequences with and without fusion to the Sf genome sequence. Furthermore, some cDNAs terminate in the middle of the open reading frame of canonical SfRV genes without employing the conserved transcription motifs reported by Ma et al. [1]. This result suggests that cDNAs containing SfRV sequence can be expressed not only by RNA dependent RNA polymerase, but also from SfRV sequence integrated in the Sf genome by the cellular transcription machinery. cDNAs found in the Bm EST library showed high similarity to the SfRV sequence (protein sequence identity = 93–99%), and a Bm mRNA clone in the nr/nt database contains a sequence with 100% identity to the SfRV genome, while Hv and Ag EST clone sequences showed low similarity to the SfRV sequence (70–80%). The presence of DNA sequences in Bm, Hy, and Ag EST that are similar or nearly identical to the SfRV sequence indicates that those sequences might represent two different rhabdovirus species that were integrated and maintained in different insect species.

The presence of two types of SfRV sequence in the Sf genome may be of evolutionary significance for this virus. Fort et al. [14] recently reported integrated rhabdovirus sequences in mosquito and other insect genomes, which represent fossil traces of the diversity of extinct rhabdoviruses. Holmes [31] commented that EVEs cease to evolve with very high substitution rates and instead replicate using high-fidelity host DNA polymerases. Fewer replications per unit time results in a dramatic reduction in evolutionary rate from virus scale to host scale, ~10^−3^ to ~10^−9^ substitutions/site/year [32]. The cDNA sequence in Sf and Bm EST clones and Sf9^L5814^ DNA derived inverse PCR clones are nearly identical to SfRV sequences. Taken with the reduced evolutionary rate, it is unlikely that SfRV replicates exogenously. Rather, an integration of the SfRV RNA sequence may have occurred in an ancestral genome prior to the divergence of Sf (Noctuidae) and Bm (Bombycidae), or the SfRV sequence may have independently integrated into the Sf and Bm genomes. Reverse transcriptase activity present in Sf cells may have been involved in the integration mechanisms [33].

Despite the common origin of the two cell lines, absence of the 320 nt sequence in total RNA is a clear difference from the SfRV sequence described by Ma et al. [1]. The deleted sequence contains a portion of the 3’ region of gene X, comprising the transcription termination motif, and the transcription initiation motif of gene L [1]. Fourteen nucleotide imperfect direct repeats may loop out the 320 nt sequence between them. The absence of adventitious viruses in insect cell lines is essential for the safety of recombinant proteins and drug products produced using the baculovirus expression system. Therefore, it would be of interest to understand how differences in maintenance and passing of Sf9 cell have led to variation of SfRV sequence between two Sf9 cell lines.

The function of the SfRV X protein, if any, remains unknown. Vasilakis et al. [15] have reported a small open reading frame, U1, between genes G and L of the two mosquito rhabdoviruses ABTV and PTAMV. U1 encodes a small hydrophobic protein, with a predicted transmembrane domain and a structural arrangement typical of class IA viroporins [22][34]. Although our bioinformatic analysis (data not shown) of the predicted translation of SfRV X gene revealed a weak prediction of a transmembrane domain for amino acid residues 40–60, typical of a class IA viroporin characteristic clusters of aromatic residues in the N-terminal ectodomain, basic residues in the C-terminal endodomain, predicted phosphorylation sites, and predicted nuclear localization signal were not evident [35]. Though similar to the N-P-M-G-U1-L structure of other rhabdoviruses, weak resemblance of the predicted SfRV X protein structure to viroporins of mononegaviruses suggests that it, if expressed, may have an alternative biological function. Moreover, C-terminal truncation may render the SfRV X gene defective or biologically inactive in Sf9^L5814^.

Sucrose density gradient purification and negative staining of the Sf9^L5814^ culture supernatant showed that the relevant fraction does not contain rhabdovirus structures or a full set of putative SfRV structural proteins. Rather, the 1.14 fraction contained amorphous membranous particles, tubular structures, a complex of the two, and a single peak containing the SfRV L gene sequence (Fig 5A). Also, the particulates seemed fragile and sensitive to EM sample preparation conditions (Fig 5B). Based on size (30 to 90 μm), morphology, and protein composition they resemble microvesicles and appear to be exosomes [36][37][38]. Our EM images are in contrast to those reported by Ma et al. [1], and these differences may be explained by an independent history of the different Sf9 cell line lots between the two laboratories.

Additionally, SDS-PAGE analysis of the 1.14 fraction did not show a typical profile of SfRV proteins. LC MS/MS analysis of the 17 major proteins indicated the presence of exosome marker protein homologs, including cytoskeletal proteins and signal transduction molecules [39], thus confirming the microvesicle-like amorphous membraneous particles as exosomes. LC MS/MS also found an incomplete set of putative SfRV M, X, L, and complete G proteins (Fig 6; S7 Table) further confirming the absence of rhabdovirus-like structures in the 1.14 fraction (Fig 5) The putative SfRV N, partial N, and P proteins in the 1.14 fraction (Fig 6; S7 Table) may serve to bind [40][41][42]and protect [43][44] the SfRV RNA sequence from nucleases in the exosome during cell to cell transport.

Diethyl ether treatment showed the SfRV L gene sequence was mostly present in the diethyl ether soluble fraction, indicating that it is not part of an enveloped virus [27][28] or quasi-enveloped virus [45]; this was in direct contrast to an extracellular baculovirus positive control. This finding is consistent with the results from our EM and LC-MS/MS analyses (Figs 5 and 6; S7 Table) indicating the absence of diethyl ether resistant viral nucleocapsid in this cell line.

Interestingly, the 1.14 fraction revealed the ability to transfer baculovirus DNA into Sf9 ^L5814^ cells. This matches the characteristic function of exosomes as transporters of nucleic acid fragments in transfection [37], further verifying that the 1.14 fraction contains exosomes. The exosome biogenesis pathway mechanism is known to overlap considerably with the assembly and egress mechanism of numerous enveloped viruses [46]. The viruses hijack host exosome pathways, and virally modified exosomes contribute to virus proliferation and immune evasion [45][47]. Their similarities suggest an evolutionary exploitation of host pathways by viruses for replication, spread, and immune evasion [47][48].

Absence of detectable SfRV particles combined with the presence of only a portion of SfRV proteins, may indicate that at some time in evolutionary history the SfRV had been an exogenously replicating virus that utilized cell to cell communication mechanisms *via* exosomes. Our data suggests that SfRV RNA was reverse transcribed and the viral DNA integrated by a reverse transcriptase involved in the machinery of cellular transposable elements [33][14][49][50][51], and therefore, transcription of the integrated and fossilized viral DNA sequence is sustained. A nearly entire SfRV L gene sequence found in contigs from the Sf9 culture supernatant may be enough to replicate itself by a mechanism similar to one employed by an exosome mediated transfer of subgenomic hepatitis C virus RNA replicon [1][52].

Our results clearly demonstrate the absence of rhabdoviral particles in ATCC CRL-1711 lot 5814 Sf9 cells in contrast to a previous study that suggested the presence of infectious rhabdoviral particles in Sf9 cells from a different lot. This study highlights how cell lines with different lineages may present different virosomes and that therefore no general conclusions can be drawn across Sf9 cells from different laboratories.

Studies to enhance our understanding on viruses endogenous to insect cells are needed. This studies would include but not be limited to transcriptome analysis of the exosome, and extensive comparative characterization of multiple cell lines with different histories and from different laboratories. Furthermore studies aimed at the mechanisms for the integration and RNA-RNA replication of SfRV RNA may address questions of how SfRV RNA sequences have become associated with hosts.

## Supporting information

S1 Table SfRV specific primers. F, forward; R, reverse on SfRV RNA.
(XLSX)

S2 Table Inverse PCR primers. F, forward; R, reverse on SfRV RNA.
(XLSX)

S3 Table qRT-PCR primers. F, forward; R, reverse.
(XLSX)

S4 Table BLASTN search of SfRV RNA sequence against SPODOBASE EST libraries. (XLSX)

S5 Table BLASTN search of SfRV RNA sequence against GenBank EST library. (XLSX)

S6 Table Sequence comparison of SfRV with RT-PCR products from Sf9 total RNA. (XLSX)

S7 Table Proteins present in 1.14 g/ml sucrose fraction of Sf9 culture supernatant identified by LC-MS/MS analysis. (XLSX)
